# The Influence of Cement Substitution by Biomass Fly Ash on the Polymer–Cement Composites Properties

**DOI:** 10.3390/ma14113079

**Published:** 2021-06-04

**Authors:** Beata Jaworska, Dominika Stańczak, Joanna Tarańska, Jerzy Jaworski

**Affiliations:** Department of Building Materials Engineering, Faculty of Civil Engineering, Warsaw University of Technology, 00-637 Warsaw, Poland; d.stanczak@il.pw.edu.pl (D.S.); joannataranska@gmail.com (J.T.); j.jaworski2@il.pw.edu.pl (J.J.)

**Keywords:** polymer–cement composites PCC, agricultural biomass fly ash, siliceous coal fly ash

## Abstract

The generation of energy for the needs of the population is currently a problem. In consideration of that, the biomass combustion process has started to be implemented as a new source of energy. The dynamic increase in the use of biomass for energy generation also resulted in the formation of waste in the form of fly ash. This paper presents an efficient way to manage this troublesome material in the polymer–cement composites (PCC), which have investigated to a lesser extent. The research outlined in this article consists of the characterization of biomass fly ash (BFA) as well as PCC containing this waste. The characteristics of PCC with BFA after 3, 7, 14, and 28 days of curing were analyzed. Our main findings are that biomass fly ash is suitable as a mineral additive in polymer–cement composites. The most interesting result is that the addition of biomass fly ash did not affect the rheological properties of the polymer–cement mortars, but it especially influenced its compressive strength. Most importantly, our findings can help prevent this byproduct from being placed in landfills, prevent the mining of new raw materials, and promote the manufacture of durable building materials.

## 1. Introduction

Increasing social consciousness and the implemented regulations demonstrate that modern society appreciates the importance of environmental problems. Importance is currently assigned to the sustainable development strategy. Sustainable development does not exclude the construction industry. In the 1980s, the World Commission on Environment and Development [1] defined the principles of sustainable development as follows: “renewable resources should not be consumed more rapidly than it is possible to reproduce; non-renewable resources should not be consumed faster than they are being replaced by renewable alternatives; and impurities and remains should not be generated faster than they can be adopted by nature, recycled, or neutralized”. Following this assumption is the essence of the overall idea that modern requirements should be met in such a way that future generations will also be able to satisfy their needs.

It is estimated that the construction industry consumes over 40% of the energy generated, about 50% of the mass of the processed materials, and emits 35% of greenhouse gases worldwide [2]. The European Regulation No. 305/2011 [3] on standardized marketing conditions of building construction, which replaced Directive 89/106/ECC [4], presents seven basic requirements for construction projects, among which there is also a sustainability requirement that a building structure should be designed, built, maintained, and demolished in accordance with the sustainable development strategy. A sustainable building fulfills the following criteria [5]: maximum reduction of energy consumption (resource conservation); maximum reusability of the elements; reconstruction; and environmental and human health protection and comfort of use (quality). The following treatments, among others, are used to balance the concrete: substitution of part of clinker in Portland cement with mineral additives (especially of waste origin); use of mineral additives for concrete production; and making concrete with lower cement content using plasticizers and superplasticizers [6]. According to the Polish Union of Byproducts of the Combustion, over 20 million tons of ashes are produced in Poland annually [7]. It is also estimated that during 2019–2024, about 2.5 MT of very high-quality fly ash will be introduced to the Polish market [8]. Reasonable management of this waste as a valuable component of cement and concretes is not only technologically justified but also eco-friendly, particularly in a country such as Poland, in which the coal power industry is still the dominant form of energy generation. However, the European Union’s requirements to reduce CO_2_ emission and increase the production of energy from renewable sources have resulted in a dynamic use of biomass for energy production in Poland. Biomass resources for the production of energy in Poland, assessed in various documents, are the highest among all other renewables. Its use is also prevalent in all sectors of power generation compared to other renewable sources. The realistic economical biomass potential in Poland was estimated at 600,168 TJ in 2020, while the market potential was estimated at 533,118 TJ according to the Institute of Renewable Energy in Poland [9]. Nevertheless, this renewable energy type as well as energy obtained from coal combustion has one major imperfection: the production of waste. Fly ash is the dominant waste residue from the coal or biomass combustion process [10,11,12]. The presence of fly ash in concrete can adversely influence the fluidity and workability of the mixtures. Increased water demand has been observed when biomass fly ash is used in concretes [13,14,15]. Cement substitution with biomass fly ash reduces the heat of hydration [16] and prevents concrete from cracking due to thermal stresses [17,18]. This substitution also affects the compressive strength of the concrete [19,20]. Wang S. et al. [21] demonstrated that biomass fly ash concrete has at least the same or considerably better parameters in strength and durability when compared to conventional fly ash concrete. Similar findings resulted from a study by Teixeira E.R. et al. [22], who reported that concrete containing biomass fly ash had a comparable carbonation resistance to conventional fly ash concrete.

Incorporation of these troublesome wastes into polymer–cement composites might be an efficient way to manage these materials. Due to the widely held view that mineral additives are poorly compatible with polymers [23], few authors have addressed the idea of introducing these additives into polymer or polymer–cement concretes [24]. The use of polymer and mineral additive in a single mixture is a very complex issue. The properties of PCC composites largely depend on the type of polymer used as well as appropriately selected curing [25]. During the hydration process, the polymer particles remain absorbed on the surface of the cement grains and mainly affect the technological characteristics, such as the viscosity and workability of the mixture [26]. In general, polymer–cement composites are characterized by higher surface adhesion, tightness, and flexural and tensile strengths compared to cement composites [25].

The possibility of using waste pearlite powder as a component of PCC was verified by Jaworska et al. [27] and the chemical resistance of such composites was evaluated in this study. The mechanical properties of PCC containing waste perlite powder were also presented by Łukowski [28]. The strength increase of polymer–cement concretes containing microsilica was confirmed by Evbuomwan [29] and Gao et al. [30], while the contribution of fly ash content to the environmental resistance of PCC was investigated by Bonora et al. [31]. Previous work by the authors on the carbonation resistance of polymer–cement composites containing biomass fly ash [32] showed that biomass fly ash increased the carbonation susceptibility of polymer–cement composites to a lower degree when compared to siliceous fly ash-containing composites.

The addition of the mineral additives gives the possibility to improve the strength and/or impermeability of the concrete and, therefore, the durability. The consequence is to minimize the necessity of repairing the concrete, which is burdensome, material-consuming, and energy-consuming, as well as improving the facility’s usage comfort. The utilization of waste products and byproducts of industrial processes, and the resulting improvement in the properties of the concrete, primarily in terms of durability, allows these modifiers to be considered sustainable materials.

Due to the harmful environmental impact, a polymer modification of the concrete may seem to be a debatable issue. However, polymer–cement composites account for a small part of the total concrete industry production. These composites, thanks to polymer modification, obtain many favorable properties, e.g., high durability, also in the sense of ensuring durability by means of repairs or structural protection. Moreover, the polymer modifier is permanently bound in the concrete and remains inside the element.

The presented research was focused on the evaluation of the application of residual fly ash (fly ash from agricultural biomass) and the influence of the quantity of such mineral additive on the strength characteristics of the polymer–cement composites at an early stage of the hardening process. The application of residual fly ash in polymer–cement composites might be an efficient way to manage these materials. The results of these investigations were combined with the results obtained for the same type of polymer–cement composition but with siliceous fly ash (SFA), a well known and studied mineral additive.

## 2. Materials and Methods

### 2.1. Materials

For the preparation of the specimens, Portland cement (CEMI 42,5R LaFarge, Małogoszcz, Poland) with high early strength and composition in accordance with the PN-EN 197-1 [33] requirements was used. As mineral additives, two types of fly ashes were used: (1) fly ash from agricultural biomass (mixture of oats and wheat), with a much higher content of such components as CaO, MgO, Na_2_O, K_2_O, and P_2_O_5_ and at the same time lower content of SiO_2_, Al_2_O_3_, and TiO_2_ as compared to siliceous coal fly ash; and (2) fly ash from EC Żerań Power Plant, Warsaw, Poland, which contains mainly SiO_2_ and Al_2_O_3_, with the content of reactive SiO_2_ is at least 25% by weight. Table 1 presents the chemical composition of the fly ashes. The density of the agricultural biomass fly ash was 2.53 g/cm^3^ and the density of the siliceous fly ash was 1.94 g/cm^3^. Siliceous fly ash was characterized by a higher content of organic fractions (5.64%) compared to biomass fly ash (3.73%). According to the PN-EN 196-2:2013-11 [34] standard, the siliceous fly ash can be classified as ash Category B (5–7% LOI) and the biomass fly ash as Category A (<5% LOI).

An aqueous dispersion of styrene-acrylic copolymer (SA) was used as a polymer modifier. This modifier is recommended to improve the properties of concrete, especially the bending tensile strength and compressive strength. The solid content of the dispersion was 33.5% and the pH of the dispersion was 7.7. Normal sand according to PN-EN 196-1 [35] with quartz content above 98% was used as aggregates.

### 2.2. Specimens Preparation

Mortars’ compositions calculated per 1 kg of the mixture are shown in Table 2. A planetary mixer was used for a total of 6 min of mixing. Seven compositions of polymer–cement composites were prepared. They differed in the type and content of fly ashes. For all compositions, the water/cement ratio (w/c = 0.5) was assumed as a constant value. Additionally, a constant polymer content (p/c = 15%) was determined. The types of mineral additive and its content we variable (m/c = 0%, 5%, 10%, and 25%).

An aqueous dispersion of the copolymer was added to the weighted and mixed dry components, as well as the remaining amount of tap water necessary to obtain the assumed water/cement ratio. After mixing all the ingredients, the mixture was transferred into the molds and covered with a foil. After 24 h, the specimens were disassembled and subjected to further treatment. PCC composites were stored in water for 5 days, and the following days in air-dry conditions. This allowed forming a polymer–cement matrix after cement hydration in the initial period of curing. The reference specimens, without polymer modifier and mineral additives, were stored in water for the whole curing time. The initial period of curing of composites was the time before 28 days from mixing the components and forming the elements. Twenty-eight days is the time in which concrete achieves most of its full strength (at 20 °C) [36,37,38,39]. To verify the strength development during this time, a sequence of testing samples after 3, 7, 14, and 28 days of curing was adopted.

### 2.3. Measurements

The research part was divided into three stages: The initial stage included qualitative research of mineral materials used. Then, the tests of the mixtures in their unhardened state were conducted such as consistency, plasticity, air content, and the beginning and the end of setting time. In the last stage, strength and carbonation tests were carried out on the hardened specimens.

The determination of the granulometric composition and the specific surface area of the mineral additives was performed using the Horiba LA-300 laser particle size analyzer (HORIBA Scientific, Kyoto, Japan. This test is based on the laser measurements of the dispersion of the laser light in the dispersion solution, in which the simultaneous determination of the average particle size in the mixture [40]. As a dispersant, a 0.1% aqueous solution of sodium polymetaphosphate PMPNa was used. The materials were analyzed at the following parameters: refraction index 1.16-0.00i, pump circulation speed 7.0 L/min, and time of application of dispersing ultrasound about 1 min. The activity index of fly ashes was determined in accordance with PN-EN 450-1 [41] standard. The consistency of the mixtures, due to their high liquidity, was carried out in Novik cone; the plasticity of the prepared composites was determined by the flow table method; and testing of air content in the unhardened mortar was carried out with the use of a pressure equalizer. All these tests were performed in accordance with PN-85/B-04500 [42]. The beginning and the end of mortar setting time was measured according to the PN-EN 196-3 [43] standard using Vicat equipment (diLUIGI GIAZZI, Bergamo, Italy). Strength tests of the specimens were measured at the age of 3, 7, 14, and 28 days in accordance with PN-85/B-04500 [42]. Carbonation resistance was measured in a carbonation chamber—in which CO_2_ concentration was kept at 1%, with a temperature of 21 ± 1 °C and a relative humidity of 60% ± 10%. Carbonation resistance was determined in accordance with the European Standard PN-EN 13295: 2005 [44]. The test was carried out on rectangular specimens with the dimensions of 40 mm × 40 mm × 160 mm after 14 and 28 days of carbonation.

## 3. Results and Discussion

### 3.1. Characteristic of the Mineral Additives

#### 3.1.1. Granulation and Specific Surface Area

**Figure 1:**
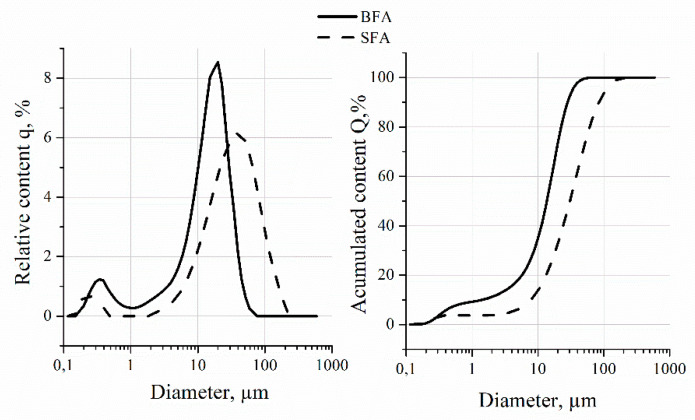
Grain size distribution relative content (**left**) and accumulated content (**right**).

The measurement results of two samples were presented in the form of granulometric curves through relative contents, q, and accumulated, Q, expressed in µm, with an accuracy of 0.01 µm; specific surface area values are expressed in cm^2^/cm^3^, with an accuracy of 1 cm^2^/cm^3^ (Figure 1). Table 3 presents the grain size distribution characteristic of the materials in the form of statistical analysis considering the mean value, coefficient of variation, median, variance, and distribution of D_50_ and D_90_ (i.e., diameters not exceeding 50% and 90% of the grains of a given material).

Siliceous fly ash was characterized by larger maximum grain size, D_max_, by more than three times compared to biomass fly ash. On the other hand, biomass fly ash was characterized by almost twice as large surface area as siliceous fly ash. Both biomass fly ash and siliceous fly ash were characterized by minimum grain size of 0.17 µm. Ninety percent of the biomass fly ash particles are smaller than 21 µm, while fractions larger than 2 mm were not recorded. Given the above and based on the grain size distribution curve analyzed for the biomass fly ash, it can be stated that the biomass fly ash is suitable for use as a substitute of cement and as a filler in polymer–cement composites.

#### 3.1.2. Activity Index of Fly Ashes

The activity index is defined as the ratio of the compressive strength of a standard mortar containing 75% by mass of the reference cement and 25% by mass of fly ash to the compressive strength of a standard mortar made with 100% of the referenced cement, expressed in a percentage. Compressive strength was determined in accordance with the PN-EN 196-1 [35] standard after 28 and 90 days of curing on three specimens for each composition. The results of the determination are presented in Table 4.

Siliceous fly ash (SFA) was characterized by AI values above 75% after 28 days and above 85% after 90 days of testing (high activity index). Agricultural biomass fly ash (BFA) was characterized by low AI after both 28 and 90 days of testing. This is due to the lower content of SiO_2_ in the BFA, which can react with calcium hydroxide creating additional C-S-H gel during cement hydration. Although biomass fly ash was characterized by lower activity index compared to siliceous fly ash still its AI was closed to 75%, and it can be stated that this ash can be used as a mineral additive in polymer–cement composites.

### 3.2. Properties of Polymer–Cement Mortars in Unhardened State

Table 5 compares the properties of polymer–cement mortars in an unhardened state.

The studies on mortars in the unhardened state were conducted to define the influence of the fly ash addition on the technological properties of the PCC composites. In the case of consistency determination with Novikov apparatus, the obtained results show a significant influence of polymer modifier addition (M0) on mortar workability—with a consistency more than double that of the reference, standardized mortar (Ref.). The cement substitution by fly ash did not affect the consistency of PCC composites regardless of the amount and type of mineral addition. The same influence of polymer modifier can be seen in the case of plasticity determination with a flow table. Here again, plasticity is more than double that of the standardized mortar, and no influence of mineral additives was observed regardless of their quantity or type. It was found that the air content of the composites is more influenced by the addition of polymer than by fly ash. Polymer–cement composites were characterized by about 80% lower air content compared to the reference mixture. No significant influence of the amount and type of mineral additive added on air content was noticed.

The properties mentioned above are strongly influenced by the properties of the materials used, such as grain size, specific surface area, and chemical compounds, as well as by the water/cement ratio. In the polymer–cement composites, the additional binder is a polymer, which affects the viscosity of the material. Additionally, in this research, a significant amount of polymer was used (15% by cement mass), so the authors expected a negligible influence of mineral additives on those properties of the composites.

**Figure 2:**
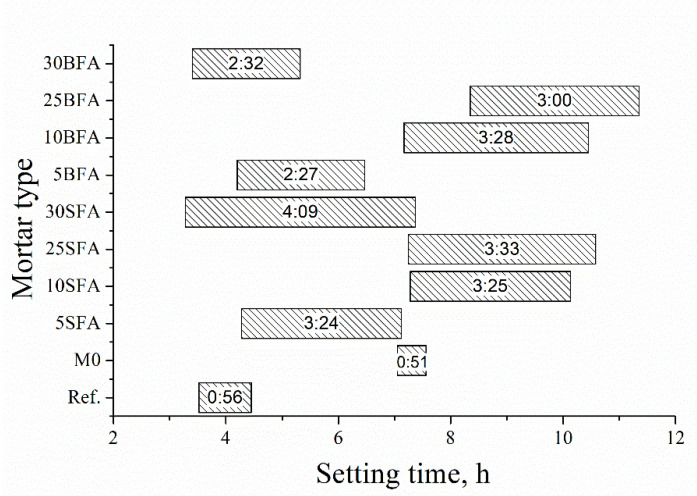
Setting time of tested mortars.

Figure 2 presents the results of the setting time measurements.

The setting time of polymer–cement composites is of significant interest for practical applications of the material. The application of 5% of fly ash, regardless of type, resulted in a significant acceleration of the beginning of setting time compared to polymer–cement mortar without mineral addition. However, the setting time of mortar containing 5% of agricultural biomass fly ash (5BFA) was shorter about 28% compared to mortar containing 5% of siliceous fly ash (5SFA). For the remaining mortars containing 10% and 25% of ashes, the setting time was comparable. The substitution of cement with a mineral additive resulted in a significant extension of mortar setting time. In the extreme cases, this extension was as much as 457% (25SFA) compared to the polymer–cement mortar without mineral additive (M0). This shows there is retardation because of the incorporation of fly ashes. The SFA is a hydraulic material and BFA is a latent hydraulic material, and, when it is introduced into the cement, its reaction must be triggered by calcium hydroxide, a cement hydration product. For this reason, it retards the cement hydration and prolongs the setting time of the composites. The initial setting delay, for the composites containing 10% and 25% of fly ashes, could be a direct result of the excessive heavy metals content [45,46,47,48]. This retardation, as reported in [49], occurs due to the transformation of metal hydroxides into new metal hydroxide compounds, resulting in considerable depletion of calcium and hydroxide ions, which significantly retards the formation of C-S-H gel and portlandite. Amphoteric metals such as zinc, lead, and tin are known to be used as setting retardants [49]. Likewise, higher loss on ignition (LOI) values can consequently cause a delay in setting [50].

### 3.3. Properties of Polymer–Cement Mortars in Hardened State

**Figure 3:**
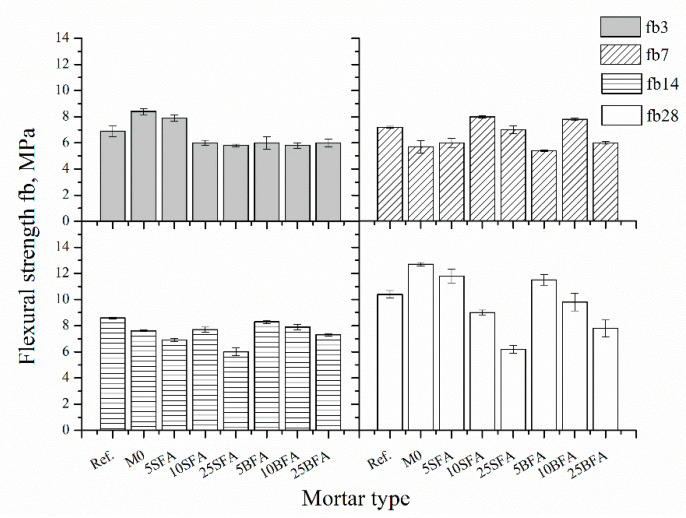
Flexural strength of tested mortars.

Flexural strength was determined according to PN-EN 196-1 [35]. A three-point loading method was used. The test was carried out on three specimens of each composition and for each term. The arithmetic mean was taken as the result. Figure 3 compares the flexural strength of tested mortars after 3, 7, 14, and 28 days of curing.

After 3 days of curing, components containing 5% of biomass fly ash (5BFA) were characterized by 25% lower flexural strength compared to their equivalents with siliceous fly ash (5SFA). Components containing more than 5% of ashes were characterized by similar flexural strength regardless of the amount and type of the ash. After 7 days of curing, the highest flexural strength was recorded for the composites containing 10% of ashes (8.0 MPa for SFA and 7.8 MPa for BFA). Polymer–cement composites with biomass fly ash, after 14 days of curing, were characterized by higher flexural strength compared to its equivalent with siliceous fly ash, even up to 18% higher for the specimens with 25% cement substitution with fly ash. The most significant influence of fly ash content on the flexural strength of tested mortars can be noticed in the case of 28-day composites. Cement substitution with fly ash up to 25% resulted in a reduction in flexural strength of polymer–cement mortars regardless of ash type; however, the reduction in flexural strength of composites containing biomass fly ash is lower compared to SFA composites. The most significant flexural strength development over time was observed for the 5SFA and 5BFA composites, with gains, respectively, of 33% and 48%. The addition of a polymer modifier to the mortar (specimens Ref. and M0) resulted in improved flexural strength. The increase in flexural strength may be due to the formation of a polymer film, which increases the strength of the binder interface between the cement hydration products and the aggregate [51]. However, by introducing mineral additives, this interface may be impaired due to either weakening of the formed polymer film or lower content of active silica in mineral additives [29].

**Figure 4:**
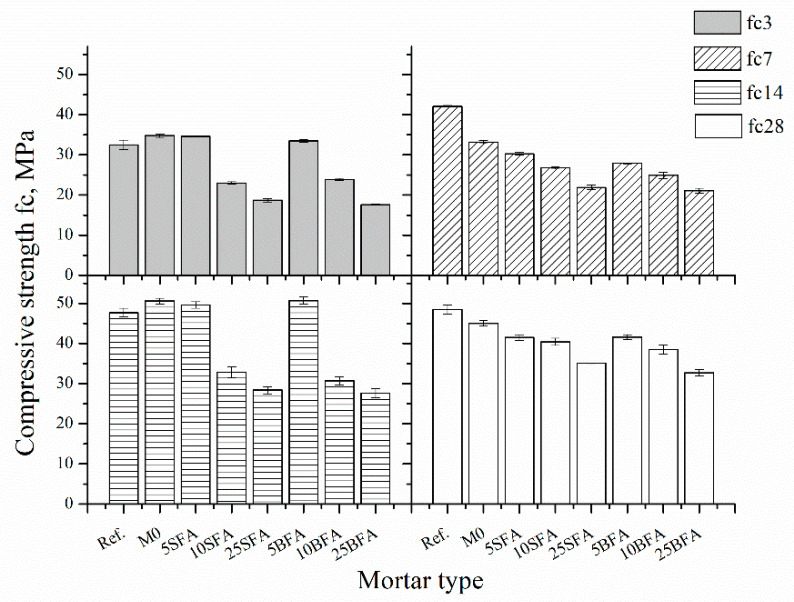
Compressive strength of tested mortars.

The compressive strength was determined in accordance with PN-EN 196-1 [35]. The test was carried out on six specimens for each composition and each term. The arithmetic mean was taken as the result. Figure 4 compares the compressive strength of tested mortars after 3, 7, 14, and 28 days of curing.

In general, as the fly ash content increases, the compressive strength of mortars decreases. The compressive strength is mostly influenced by the bonding forces generated by the hydration reaction of cement. Here, composites contain not only cement but also polymer modifier, which forms a polymer–cement matrix. The dispersion action on flocculated cement particles retards the concentration of contact points between different grains of cement, due to which the compressive strength of PCC is lower compared to unmodified cement mortar. Additionally, the lower activity index of mineral additives (compared to Cement AI) used further reduced the compressive strength of tested components. This is in agreement with reported research which concluded that the reduction in compressive strength could be due to both the decelerating effect of polymer modifiers on cement hydration and the volume change of the mortar [51].

After 3 days of curing, components containing 5% of ashes (5SFA and 5BFA) were characterized by the same compressive strength compared to M0 composites. The addition of fly ashes up to 25% reduced the compressive strength of the composites by almost two times in relation to PCC composites without mineral additive (M0). The same was noted for the composites after 14 days of curing. Here again, 5% addition of fly ash had a negligible effect on the compressive strength of the composites, and composites containing 25% of ashes were characterized by two times lower compressive strength compared to M0 composites. The compressive strength of PCC composites decreased linearly reaching the values of 21.9 and 35.1 MPa for 25SFA and 21.1 and 32.7 MPa for 25BFA, respectively, after 7 and 28 days of curing. The most significant compressive strength development over time was observed for the 25SFA and 25BFA composites, with gains, respectively, of 47% and 46%.

**Figure 5:**
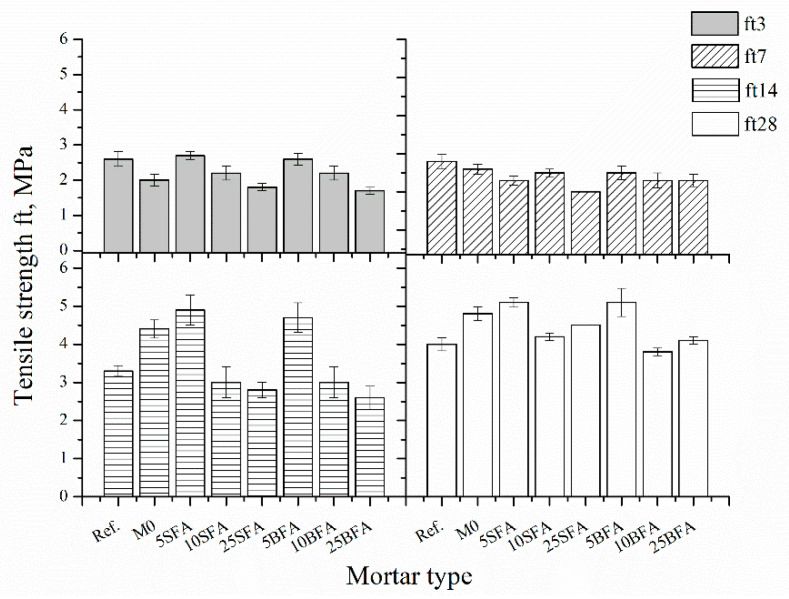
Tensile strength of tested mortars.

Tensile strength was determined in accordance with the PN-B-85-04500 [42] standard. The test was carried out on three specimens for each composition and for each term. The arithmetic mean was taken as the result. Figure 5 compares the tensile strength of tested mortars after 3, 7, 14, and 28 days of curing.

After 3 days of curing, PCC composites with mineral additives were characterized by the same tensile strength regardless of the fly ash type. After 7 days of curing, all PCC composites were characterized by a similar tensile strength except for 25SFA components, for which the tensile strength was 13% lower compared to 25BFA. The most significant influence of fly ash content on the tensile strength of tested mortars can be noticed in the case of 14-day composites. Composites containing 5% of ashes were characterized by the highest tensile strength, even higher compared to both unmodified standardized cement mortar (Ref.) and unmodified PCC mortar (M0). This tendency was maintained in the case of 28-day composites, where again composites with 5% of ashes were characterized by the highest tensile strength. The most significant tensile strength development over time was observed for the 25SFA and 25BFA composites, with gains, respectively, of 60% and 59%. The addition of polymer modifier to the mortar (Specimens Ref. and M0) resulted in improved tensile strength. The improvement in tensile strength was found to mainly depend on the polymer content. This is related to the superior tensile performance of the polymer film that creates the polymer–cement matrix [51].

The elongation of the specimens by moving the measuring beam of the testing machine was recorded simultaneously with the tensile strength test. Table 6 compares the elongation at break of tested mortars after 3, 7, 14, and 28 days of curing. The elongation values are expressed in percent with an accuracy of 0.1%.

The conducted measurements did not show any significant influence of mineral additives on the elongation change at the break of polymer–cement composites. The largest increase in elongation was observed for the polymer–cement composite with 5% substitution of siliceous fly ash (5SFA) after 28 days of curing. The lowest elongation was recorded for PCC with 5% cement substitution with siliceous fly ash (5SFA) after 3 days of curing. This property of the material, as it was for the properties of mortars in an unhardened state, was more influenced by the presence of polymer modifier than fly ashes. The obtained results do not show a significant influence of the type and quantity of the ash used on the elongation at break of tested mortars.

Table 7 shows the carbonation depth of tested mortars after 14 and 28 days of carbonation.

As observed in Table 7, polymer–cement composites are characterized by higher carbonation depth compared to standardized mortar (Ref.), being 85% and 52% higher, respectively, after 14 and 28 days of carbonation. The increased amount of fly ash in the PCC composites resulted in an increase in the depth of carbonation. Specimens containing 25% of fly ashes were characterized by the lowest resistance to carbonation. For 25BFA specimens, carbonation depth was 76% and 109% higher and for 25SFA specimens was 65% and 128% higher in relation to M0 specimens after 14 and 28 days of carbonation, respectively. In addition, the longer was the exposure to gaseous CO_2_, the lower was the observed resistance to carbonation of the specimens. The diffusion capability of CO_2_ deep into the concrete matrix depends on the concrete’s impermeability, which is influenced by such factors as the water–cement relation and production conditions. The intensity of this process has a substantial influence on the progression rate of the carbonation front into the structure of the concrete. Compact concrete, with impeded CO_2_ gas diffusion and a limited number of capillary pores, makes the CO_2_ gas-phase contact area with those capillaries relatively small, resulting in a slow carbonation process. Fly ash used in cement concretes reduces the amount of calcium hydroxide needed for CO_2_ binding and causes an increase in the capillary pore content, making such concretes more susceptible to carbonation. The case is slightly different for polymer–cement composites. For PCC, besides the proper curing procedure, the duration of component exposition to CO_2_, or the composites age, the important factors that affect the rate of carbonation process are the polymer modifier type and its quantity. With the increasing polymer content in polymer–cement composites, the carbonation rate increases, reaching maximum values at polymer content of 10–15% [52]. Despite 80% lower air content in mortars in the unhardened state (Specimens Ref. and M0; Table 5), the polymer–cement composites were characterized by reduced resistance to carbonation. This may indicate that pores were formed in the binder microstructure during curing, which increased the total porosity of the PCC mortars in comparison to the reference, cement mortar. The increase in porosity may be due to the decelerating effect of the polymer on cement hydration or the change in mortar volume [51]. In addition, the introduction of mineral additives with irregular shapes may also contribute to the increase in the porosity of mortars and therefore to the reduction in resistance to carbonation [11].

### 3.4. Concrete Early Strength Development

**Figure 6:**
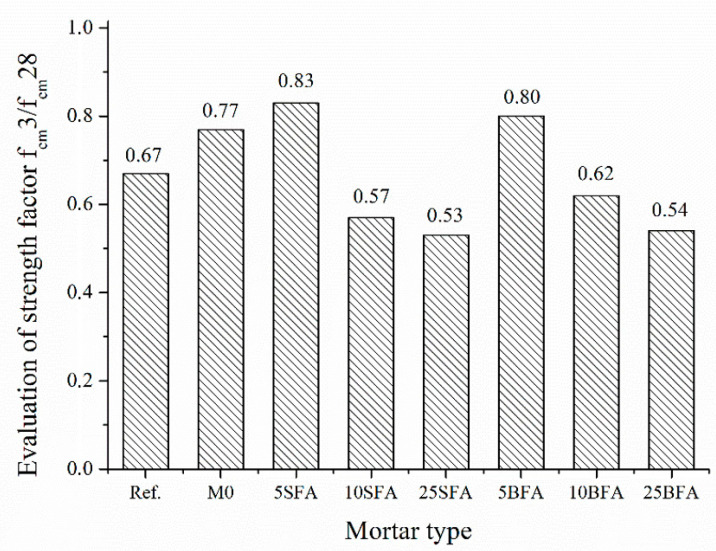
Early strength development of tested mortars.

The concrete early strength development is determined by the ratio of the average 2-day compressive strength to the average 28-day compressive strength. However, this applies to cement mortars and concretes. For the polymer–cement composites tested, it was impossible to determine the compressive strength after 2 days of curing, due to the insufficient hardness of these mortars during this time. Therefore, it was decided to evaluate the strength development only after 3 days of curing. Concrete early strength development according to PN-EN 206 [53] is presented in Table 8. Figure 6 compares the early strength development of tested mortars.

The determined modified early strength coefficients of the studied composited were compared with those in Table 8. All composites were characterized by the fast development of early strength (>0.5). Composites containing 5% of ashes were characterized by the highest modified early strength factor f_cm_3/f_cm_28. The slowest increase in strength was observed in the case of polymer–cement composites with 25% addition of siliceous fly ash (f_cm_3/f_cm_28 = 0.53).

## 4. Conclusions

The obtained results lead to several conclusions, which tend to confirm the hypothesis that agricultural biomass fly ash is useful as a mineral additive in polymer–cement composites. Consequently, we proceed to identify the following conclusions:

- The grading and activity index measurements of the agricultural biomass fly ash confirmed the possibility of using it as a mineral additive in polymer–cement composites.
- The addition of fly ash in polymer–cement composites retards cement hydration and extends the setting time of composites, but it has negligible influence on the properties of the mortars in an unhardened state.
- The properties of polymer–cement composites in the hardened state are strongly influenced by mineral additives used, especially in the case of compressive strength.
- The increased amount of fly ash in the PCC composites resulted in an increase in the depth of carbonation regardless of the type and amount of fly ash. In addition, the longer is the exposure to the gaseous CO_2_, the lower is the observed resistance to carbonation of the specimens.
- All composites were characterized by fast development of early strength (>0.5).

Derived from these conclusions, it can be stated that agricultural biomass fly ash can be used as a partial cement substitute in polymer–cement composites. In addition, its use as a mineral additive in PCC composites develops composites with similar properties to PCC composites with siliceous fly ash, a well known and studied mineral additive. It should also be pointed out that the use of a waste product that is at this stage unused in the PCC composites prevents this byproduct from being placed in landfills, prevents the mining of new raw materials, and manufactures durable building materials. Further studies related, among others, to the microstructure of PCC composites with biomass fly ash should be conducted to further evaluate the phenomena affecting the compatibility of the discussed materials.

## Figures and Tables

**Table 1:** Chemical composition of fly ashes used.

	SiO_2_	Fe_2_O_3_	Al_2_O_3_	TiO_2_	CaO	MgO	P_2_O_5_	Na_2_O	K_2_O	Others
BFA	25.8	0.5	8.5	0.3	15.2	9.5	6.2	7.4	8.5	18.1
SFA	51.6	6.6	22.9	0.9	3.8	2.2	0.6	0.7	1.9	8.8

**Table 2:** Material composition of designed mortars per 1 kg of mixture.

Symbol	Polymer Content, %	Mineral Additive Content, %	Cement, g	Water, g	Sand, g	Dispersion, g	Siliceous Fly ash, g	Biomass Fly ash, g
Ref.	-	-	222.0	111.0	667.0	-	-	-
M0	15	-	215.0	92.0	645.0	48.0	-	-
5SFA	15	5	204.0	92.0	645.0	48.0	11.0	-
10SFA	15	10	194.0	92.0	645.0	48.0	22.0	-
25SFA	15	25	161.0	92.0	645.0	48.0	54.0	-
5BFA	15	5	204.0	92.0	645.0	48.0	-	11.0
10BFA	15	10	194.0	92.0	645.0	48.0	-	22.0
25BFA	15	25	161.0	92.0	645.0	48.0	-	54.0

**Table 3:** Statistical analysis of grain size distribution of the mineral additives.

Properties	Average, µm	Median, µm	Varian, µm^2^	CV, %	Mode, µm	D_min_, µm	D_max_, µm	D_50_, µm	D_90_, µm	S.P., cm^2^/cm^3^
SFA	39.6	30.6	1087.0	83.2	36.6	0.17	200.0	29.9	77.4	12755
BFA	14.7	13.7	101.8	68.2	16.5	0.17	67.5	13.3	26.1	22280

**Table 4:** Activity index of tested fly ashes.

Composition	f_c,28_, MPa	AI_28_	f_c,90_, MPa	AI_90_
Ref.	44.4	-	50.8	-
SFA	42.7	96.2	54.2	106.8
BFA	32.1	72.3	36.6	72.0

**Table 5:** Properties of polymer–cement mortars in unhardened state.

Symbol	Consistency, cm	Plasticity, cm	Air Content, %
Ref.	5.5	13.5	6.4
M0	11.3	26.5	1.2
5SFA	11.5	25.5	0.7
10SFA	11.4	26.5	1.1
25SFA	11.3	22.6	1.2
5BFA	11.5	29.5	1.3
10BFA	11.5	28.4	1.8
25BFA	11.5	29.2	1.9

**Table 6:** Elongation at break of tested mortars.

Symbol	Δl3, %	Δl7, %	Δl14, %	Δl28, %
Ref.	1.2	1.2	1.3	1.6
M0	1.2	1.3	1.3	1.8
5SFA	1.0	1.6	1.5	1.9
10SFA	1.5	1.6	1.3	1.6
25SFA	1.7	1.5	1.8	1.8
5BFA	1.1	1.5	1.4	1.5
10BFA	1.3	1.5	1.4	1.6
25BFA	1.2	1.4	1.4	1.7

**Table 7:** Carbonation depth of tested mortars.

Symbol	d_k_14, mm	d_k_28, mm
Ref.	1.4	2.3
M0	2.6	3.5
5SFA	3.4	4.1
10SFA	3.2	5.8
25SFA	4.3	8.0
5BFA	3.3	4.4
10BFA	3.6	5.5
25BFA	4.6	7.3

**Table 8:** Concrete early strength development according to PN-EN 206 [53].

Development of Concrete Strength	Evaluation of Strength Factor f_cm_2/f_cm_28
Fast	≥0.5
Moderate	≥0.3 to<0.5
Slow	≥0.15 to <0.3
Very slow	<0.15

## Data Availability

The data presented in this study are available on request from the corresponding author.

## References

[1] Imperatives S. (1987). Report of the World Commission on Environment and Development: Our common future. Accessed Feb.

[2] Czarnecki L., Kaproń M., Piasecki M., Wall S. (2012). Sustainable construction is the construction of the future—In polish. Inżynieria I Bud..

[3] European Commission (2011). Regulation (EU) No 305/2011 of the European Parliament and of the Council of 9 March 2011 laying down harmonized conditions for the marketing of construction products and repealing Council Directive 89/106/EEC. Off. J. Eur. Union.

[4] Council Directive (1988). 89/106/EEC Council Directive 89/106. EEC of 21 December 1988 on the approximation of laws, regulations and administrative provisions of the Member States relating to construction products. Off. J. Eur. Union.

[5] Kibert C.J. Establishing principles and a model for sustainable construction. Proceedings of the First International Conference on Sustainable Construction.

[6] Czarnecki L., Justnes H. (2012). Sustainable & durable concrete. Cem. Wapno Beton.

[7] Chrzanowski Z., Masłowski D. (2014). Utilization of combustion by-products in Poland—In polish. Mater. Bud..

[8] Chrzanowski Z., Baran B., Dudziak M., Katzor R. Poland as a potential source of combustion by-products (UPS) for European markets—In polish. Proceedings of the XXVI Międzynarodowa Konferencja Popioły z Energetyki.

[9] (2007). Possibilities of Using Renewable Energy Sources in Poland 2020.

[10] Melotti R., Santagata E., Bassani M., Salvo M., Rizzo S. (2013). A preliminary investigation into the physical and chemical properties of biomass ashes used as aggregate fillers for bituminous mixtures. Waste Manag..

[11] Ohenoja K., Pesonen J., Yliniemi J., Illikainen M. (2020). Utilization of fly ashes from fluidized bed combustion: A review. Sustainability.

[12] Nunes L., Matias J., Catalão J. (2016). Biomass combustion systems: A review on the physical and chemical properties of the ashes. Renew. Sustain. Energy Rev..

[13] Wang S., Miller A., Llamazos E., Fonseca F., Baxter L. (2008). Biomass fly ash in concrete: Mixture proportioning and mechanical properties. Fuel.

[14] (2002). Demonstration of Manufacturing Technology for Concrete and CLSM Utilizing Wood Ash from Wisconsin.

[15] Elinwa A.U., Mahmood Y.A. (2002). Ash from timber waste as cement replacement material. Cem. Concr. Compos..

[16] Popławski J., Lelusz M. (2017). Utility assessment of biomass fly-ash for production of concrete products. Czas. Tech..

[17] Lindon K., Sear A. (2001). Properties and Use of Coal Fly Ash. A Valuable Industrial by-Product.

[18] Thomas M. (2007). Optimizing the Use of Fly Ash in Concrete.

[19] Lutze D., Berg W. (2010). Popiół lotny w betonie. Poradnik.

[20] Pera J., Ambroise J. Influence of different polymers on the properties of cementitious matrices. In Proceedings of the 10th International Congress on Polymers in Concrete—ICPIC’01.

[21] Wang S., Baxter L. (2007). Comprehensive study of biomass fly ash in concrete: Strength, microscopy, kinetics and durability. Fuel Process. Technol..

[22] Teixeira E.R., Camões A., Branco F., Aguiar J., Fangueiro R. (2019). Recycling of biomass and coal fly ash as cement replacement material and its effect on hydration and carbonation of concrete. Waste Manag..

[23] Ozkul M. Effect of aggregate on the properties of epoxy concrete. Proceedings of the 8th International Congress on Polymers in Concrete.

[24] Gorninski J.P., Dal Molin D.C., Kazmierczak C.S. (2004). Study of the modulus of elasticity of polymer concrete compounds and comparative assessment of polymer concrete and portland cement concrete. Cem. Concr. Res..

[25] Łukowski P. (2008). Rola polimerów w kształtowaniu właściwości spoiw i kompozytów Polimerowo-Cementowych.

[26] Betioli A.M., Gleize P.J.P., John V.M., Pileggi R.G. (2012). Effect of EVA on the fresh properties of cement paste. Cem. Concr. Compos..

[27] Jaworska B., Sokołowska J., Łukowski P., Jaworski J. (2015). Waste mineral powders as a components of polymer-cement composites. Arch. Civ. Eng..

[28] Łukowski P. (2016). Polymer-cement composites containing waste perlite powder. Materials.

[29] Evbuomwan N. Strengtening effects of microsilica in a polymer modified mortar under different curing regimes. Proceedings of the 3rd Southern African Conference on Polymers in Concrete.

[30] Gao J., Qian C., Wang B., Morino K. (2002). Experimental study on properties of polymer-modified cement mortars with silica fume. Cem. Concr. Res..

[31] Bonora V.S.A., Sandrolini F., Belz G., Dinelli G. Resistance to environmental attack of polymer modified mortars containing fly ashes. Proceedings of the International Congress on Polymers in Concrete.

[32] Stańczak D., Jaworska B. (2020). Influence of agricultural biomass fly ash cement substitution on the carbonation of cement and polymer-cement composites. Struct. Environ..

[33] (2012). PN-EN 197-1: 2012 Cement—Part 1: Composition, Specifications and Conformity Criteria for Commercial Cements.

[34] (2013). PN-EN 196-2:2013-11 Method of Testing Cement—Part 2: Chemical Analysis of Cement.

[35] (2018). PN-EN 196-1 Cement Testing Methods. Part 1: Determination of Strength.

[36] Ejeh S., Uche O. (2009). Effect of crude oil spill on compressive strength of concrete materials. J. Appl. Sci. Res..

[37] Gartner E., Quillin K. (2007). Low-CO_2_ cements based on calcium sulfoaluminates. Sustain. Cem. Concr. Ind. Nor. Cem. Assoc. Sept..

[38] Hasan M., Kabir A. (2013). Early age tests to predict 28 days compressive strength of concrete. Casp. J. Appl. Sci. Res..

[39] Lee C., Lee S., Nguyen N. (2016). Modeling of compressive strength development of high-early-strength-concrete at different curing temperatures. Int. J. Concr. Struct. Mater..

[40] Bohren C.F., Huffman D.R. (2008). Absorption and Scattering of Light by Small Particles.

[41] (2012). PN-EN 450-1:2012 Fly Ash for Concrete. Part 1: Definitions, Specifications and Conformity Criteria.

[42] (1985). PN-85/B-04500 Construction Mortars. Research of Physical and Strength Characteristics.

[43] (2018). PN-EN 196-3 Cement Testing Methods. Part 3: Determination of Setting Times and Volume Stability.

[44] (2005). PN-EN 13295: 2005 Products and Systems for the Protection and Repair of Concrete Structures—Test Methods—Determination of Resistance to Carbonation.

[45] Stephan D., Mallmann R., Knöfel D., Härdtl R. (1999). High intakes of Cr, Ni, and Zn in clinker: Part I. Influence on burning process and formation of phases. Cem. Concr. Res..

[46] Olmo I.F., Chacon E., Irabien A. (2001). Influence of lead, zinc, iron (III) and chromium (III) oxides on the setting time and strength development of Portland cement. Cem. Concr. Res..

[47] Rossetti V., Medici F. (1995). Inertization of toxic metals in cement matrices: Effects on hydration, setting and hardening. Cem. Concr. Res..

[48] Rai A., Prabakar J., Raju C., Morchalle R. (2002). Metallurgical slag as a component in blended cement. Constr. Build. Mater..

[49] Weeks C., Hand R.J., Sharp J.H. (2008). Retardation of cement hydration caused by heavy metals present in ISF slag used as aggregate. Cem. Concr. Compos..

[50] Chen H.-J., Shih N.-H., Wu C.-H., Lin S.-K. (2019). Effects of the loss on ignition of fly ash on the properties of high-volume fly ash concrete. Sustainability.

[51] Li P., Lu W., An X., Zhou L., Du S. (2021). Effect of Epoxy Latexes on the Mechanical Behavior and Porosity Property of Cement Mortar with Different Degrees of Hydration and Polymerization. Materials.

[52] Woliński P., Woyciechowski P., Jaworska B., Adamczewski G., Tokarski D., Grudniewski T., Chodyka M., Nitychoruk J.A. (2018). The influence of the mineral additives on the carbonation of cement composites. MATEC Web of Conferences.

[53] (2017). PN-EN 206+A1:2016-12 Concrete—Requirements, Properties, Production and Conformity.

